# Hyperoxia does not affect oxygen delivery in healthy volunteers while causing a decrease in sublingual perfusion

**DOI:** 10.1111/micc.12433

**Published:** 2018-02-09

**Authors:** Bob Smit, Yvo M. Smulders, Etto C. Eringa, Harry P. M. M. Gelissen, Armand R. J. Girbes, Harm‐Jan S. de Grooth, Hans H. M. Schotman, Peter G. Scheffer, Heleen M. Oudemans‐van Straaten, Angelique M. E. Spoelstra‐de Man

**Affiliations:** ^1^ Department of Intensive Care VU University Medical Center Amsterdam The Netherlands; ^2^ Department of Internal Medicine VU University Medical Center Amsterdam The Netherlands; ^3^ Department of Physiology VU University Medical Center Amsterdam The Netherlands; ^4^ Department of Clinical Chemistry VU University Medical Center Amsterdam The Netherlands

**Keywords:** dose response, healthy volunteers, hyperoxia, microcirculation, oxygen, oxygen delivery

## Abstract

**Objective:**

To determine the human dose‐response relationship between a stepwise increase in arterial oxygen tension and its associated changes in DO_2_ and sublingual microcirculatory perfusion.

**Methods:**

Fifteen healthy volunteers breathed increasing oxygen fractions for 10 minutes to reach arterial oxygen tensions of baseline (breathing air), 20, 40, 60 kPa, and max kPa (breathing oxygen). Systemic hemodynamics were measured continuously by the volume‐clamp method. At the end of each period, the sublingual microcirculation was assessed by SDF.

**Results:**

Systemic DO_2_ was unchanged throughout the study (*P*_slope_ = .8). PVD decreased in a sigmoidal fashion (max −15% while breathing oxygen, SD18, *P*_slope_ = .001). CI decreased linearly (max −10%, SD10, *P*_slope_ < .001) due to a reduction in HR (max −10%, SD7, *P*_slope_ = .009). There were no changes in stroke volume or MAP. Most changes became apparent above an arterial oxygen tension of 20 kPa.

**Conclusions:**

In healthy volunteers, supraphysiological arterial oxygen tensions have no effect on systemic DO_2_. Sublingual microcirculatory PVD decreased in a dose‐dependent fashion. All hemodynamic changes appear negligible up to an arterial oxygen tension of 20 kPa.

**Abbreviations**
BSA: body surface area
C_a_O_2_: arterial oxygen content
CI: cardiac index
DO_2_: oxygen delivery
F_I_O_2_: fraction of inspired oxygen
Hb: hemoglobin
HR: heart rate
ICU: intensive care unit
IFD: intermittent flow density
MAP: mean arterial pressure
MFI: microvascular flow index
P_a_O_2_: arterial partial pressure of oxygen
PPV: proportion of perfused vessels
PVD: perfused vessel density
S_a_O_2_: arterial oxygen saturation
SDF: sidestream darkfield imaging
SVI: stroke volume index
SVR: systemic vascular resistance
SVRI: systemic vascular resistance index
VD: vessel density

## 1 INTRODUCTION

Supplemental oxygen is administered to patients with arterial hypoxemia to ensure sufficient DO_2_ to organs. However, in clinical practice, physicians are inclined to administer oxygen profusely, even in patients who are not hypoxemic.^1^, ^2^, ^3^ As a result, supraphysiological oxygen tensions (hyperoxia) are frequently encountered.^4^ Restoring normal arterial oxygen tensions (P_a_O_2_) is obviously beneficial in hypoxemic patients, but it is uncertain whether oxygen supplementation beyond normoxia is safe and actually improves DO_2_.

Hyperoxia may increase ICU mortality^5^, ^6^, ^7^ and myocardial infarct size.^8^ On the other hand, moderate hyperoxia may alleviate organ dysfunction after cardiac arrest.^9^ In mechanically ventilated ICU patients, the (retrospective) relation between the degree of hyperoxia and mortality is U‐shaped, with a nadir around 15‐20 kPa.^10^ Potential adverse effects of hyperoxia may occur via microvascular constriction^11^, ^12^ and a reduction in cardiac output.^13^, ^14^, ^15^, ^16^ However, findings regarding such effects are ambiguous.^17^, ^18^ The reduced perfusion and cardiac output may lead to a net loss of DO_2_ that had been found in some,^19^, ^20^, ^21^ but not all studies.^22^, ^23^, ^24^

The evidence for hyperoxia causing microvascular constriction mostly comes from animal studies. In humans, the effects of hyperoxia on the microvasculature consists of indirect measures, such as an increase in SVR^15^, ^25^, ^26^, ^27^ or a reduction in peripheral blood flow.^28^, ^29^, ^30^, ^31^ Recently, a direct effect of hyperoxia on the sublingual microcirculation was shown.^32^ In this study, a marked decrease in PVD (−30%) was observed, when 10 healthy volunteers breathed pure oxygen for 30 minutes. However, as with most studies on hyperoxia, only 2 inspired oxygen concentrations were studied; air (21% O_2_) and pure oxygen (100%). Although this comparison creates the highest contrast, its clinical relevancy is limited. An F_I_O_2_ of 1.0 is rarely used in daily practice to avoid the direct toxicity of pure oxygen to the lungs. Second, the P_a_O_2_s that arise from breathing pure oxygen by healthy volunteers is not comparable to the ones in patients with existing lung pathology. As a result, the relation between hemodynamic effects of oxygen and P_a_O_2_ at clinically relevant doses remains unknown.

Only a few groups investigated the dose‐response effect of oxygen on the cardiovascular system^15^, ^26^ and none directly visualized the microcirculation. It is therefore currently unknown at which P_a_O_2_ the microcirculatory effects of hyperoxia start to occur and what the nature of the dose‐response effect is.

The aim of this study was to determine the dose‐response relationship between a stepwise increase in P_a_O_2_ and its associated changes in DO_2_ and sublingual microcirculatory perfusion.

## 2 MATERIALS AND METHODS

### 2.1 Study design and ethical approval

Single‐blind, cross‐over physiological study with healthy volunteers performed at the ICU of the VU University Medical Centre (Amsterdam, the Netherlands). The study protocol was approved by the Dutch Central Committee on Research Involving Human Subjects (NL5816602916) and conformed to the standards set by the Declaration of Helsinki.

### 2.2 Subjects

Volunteers were recruited through social media and were eligible for participation if they were 18 years or older and had no medical history of pulmonary or cardiovascular disease. A modified Allen test was performed to assess arterial competency, and subjects without a patent ulnar artery were not included. Subjects were included after written informed consent was obtained.

### 2.3 Protocol

#### 2.3.1 Preparation

Subjects lay in a semirecumbent position in a temperature‐controlled room at the ICU. After application of a local anesthetic (lidocaine), the radial artery was cannulated for blood sampling and blood pressure measurements. A finger cuff was placed on the index or middle finger for continuous measurement of hemodynamic parameters by the volume‐clamp method, according to the manufacturer's instructions (Nexfin^®^, BMEYE, Amsterdam, the Netherlands). Finally, subjects were fitted with a noninvasive ventilation mask coupled to a SERVO‐I mechanical ventilator (Maquet, Rastatt, Germany). The ventilator was set to provide zero continuous positive airway pressure or pressure support. When the subjects were accustomed to the setup (~15 minutes after radial artery cannulation), the intervention and measurements were started.

#### 2.3.2 Intervention

The F_I_O_2_ was adjusted to reach target P_a_O_2_s of baseline (kPa while breathing air), 20, 40, 60 kPa, and max kPa (while breathing pure oxygen) during 5 separate phases. Five minutes into each phase, arterial blood gas analysis was performed and the F_I_O_2_ was adjusted once if P_a_O_2_ was not at the intended target. After an additional 5 minutes, a second arterial blood gas was taken. When all study measurements were performed (see below), the subject rested 5‐10 minutes before moving on to the next P_a_O_2_ target. Subjects knew they would inspire F_I_O_2_s between 21%‐100%, but were unaware of the predetermined stepwise increase. Monitors and the control of F_I_O_2_ were not visible for the participants.

### 2.4 Measurements

At the end of each period, the NIV mask was removed and the sublingual microcirculation was visualized immediately (within one minute) with SDF (MicroVision Medical BV, Amsterdam, the Netherlands). In SDF imaging, green light is emitted from the device which is then absorbed by the Hb present in erythrocytes. SDF therefore relies on the presence of Hb to visualize blood vessels. Three to 5 sites were recorded and analyzed in accordance with the latest quality recommendations.^33^ After acquisition, the video files were stored for blinded offline semiquantitative analysis with the Automated Vascular Analysis software 3.1 (MicroVision Medical BV). In short, a grid of 5 equidistant vertical and horizontal lines is placed on top of the recording. Vessels crossing these lines are counted and classified as having either continuous, slow/sluggish, intermittent, or no flow. Vascular density (VD) is reported as the total number of vessels per mm of grid. PVD is comprised of vessels showing only continuous or slow/sluggish flow. Although not regularly reported, we also calculated the number of intermittent perfused vessels (IFD) in a similar fashion. All recordings and analyses were carried out by the same operator (BS). All data reported pertain to small vessels with a diameter of 20 μm or less.

Heart rate, CI, SVI, and SVRI were measured continuously during the entire experiment. MAP was measured via the arterial line. The average of the last 2 minutes of each exposure was used for statistics.

Blood gas and metabolite parameters were measured on‐site with an ABL800 FLEX analyzer (Radiometer, Copenhagen, Denmark).

### 2.5 Calculations

Systemic DO_2_ index was calculated by multiplying CI with the C_a_O_2_. For the latter, the following formula was used; C_a_O_2_ = (Hb [g/dL] × 10 × 1.36 × S_a_O_2_) + (0.0031 × [P_a_O_2_ (kPa) × 7.5]).

### 2.6 Statistics

All values are reported as mean and standard deviation unless stated otherwise. Dose‐response relations were primarily fitted with a linear regression based on the parameters at each kPa target. An additional nonlinear regression was performed if deemed warranted based on visual inspection. Fit performance was assessed visually and by means of the Sy.x statistic (standard deviation of errors in regression). For all data, we tested whether the slope was statistically different from zero. All graphs and statistics were carried out with GraphPad Prism 7.0 (GraphPad Software, Inc., La Jolla, CA, USA).

## 3 RESULTS

### 3.1 Volunteers and measurements

Baseline characteristics of the 15 included volunteers are listed in Table 1. All participants gave written informed consent and completed the entire protocol without adverse events. On average, the duration of the study was 95 minutes (SD 8). In one subject, continuous measurement of hemodynamic parameters by the volume‐clamp method was omitted because a stable, valid waveform could not be obtained (due to peripheral vasoconstriction). Sublingual measurements and MAP values obtained through the arterial line did not differ from the other participants. Therefore, the sublingual data of this participant were included in the final analysis.

**Table 1:** Baseline characteristics

Variable	Participants
Number	15
Gender (male/female)	7/8
Age (y)	30 (9)
Height (cm)	175 (9)
Weight (kg)	71 (11)
BSA (m^2^)	1.85 (0.21)

BSA, body surface area.

### 3.2 Intervention/Respiration/Blood gas

The obtained P_a_O_2_s during the 5 periods were 14 kPa (SD 1.3), 20 kPa (SD 1.8), 39 kPa (SD 3.0), 57 kPa (SD 4.9), and 73 kPa (SD 4.1). The F_I_O_2_s required to reach these oxygen tensions was 21% (SD 0), 29 (SD 2), 55 (SD 3), 80 (SD 5), and 100 (SD 0), respectively. Blood gas analysis revealed no changes in P_a_CO_2_ or pH. Glucose and lactate decreased slightly during the study period but remained within normal ranges.

### 3.3 Systemic hemodynamic effects

**Figure 1:**
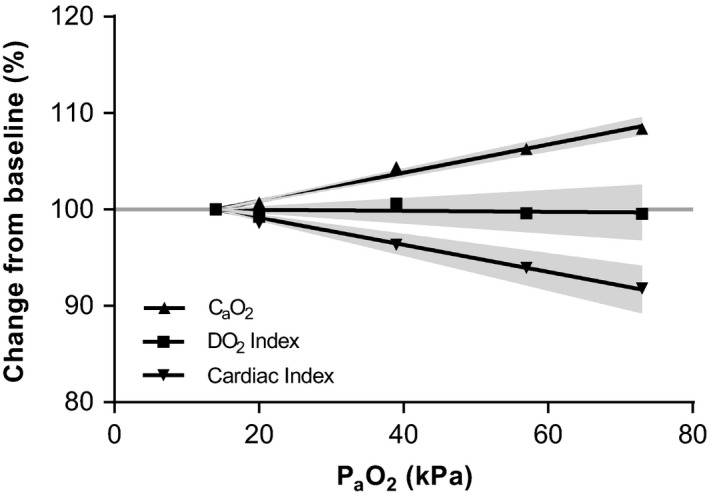
Relation between oxygen content, delivery, and CI. Oxygen content increased linearly with increasing P_a_O_2_. Inversely, CI decreased, which resulted in a stable DO_2_I over the entire P_a_O_2_ range. Gray areas indicate 95% confidence intervals of the fitted curve. P_a_O_2_, arterial oxygen tension; C_a_O_2_, arterial oxygen content; DO_2_I, arterial oxygen delivery index; CI, Cardiac index

**Figure 2:**
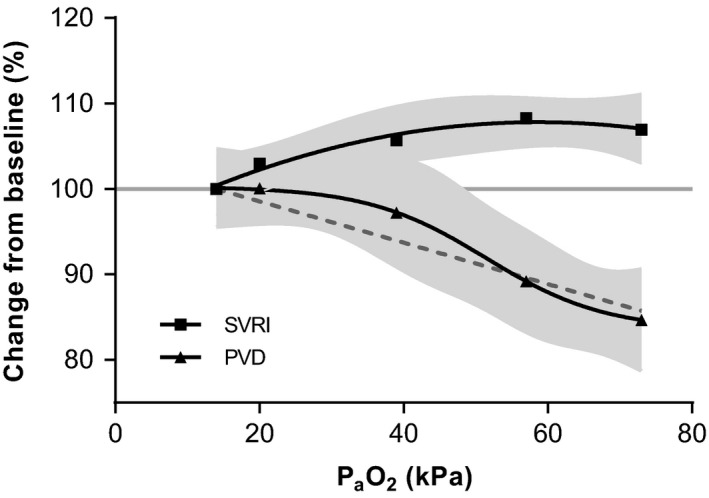
Dose‐response for PVD and SVRI. Sublingual PVD decreases in a sigmoidal fashion upon increase P_a_O_2_. SVRI shows the largest increase up to 54 kPa. Gray areas indicate 95% confidence intervals of the fitted curves. For PVD, the dotted line represents the best‐fit line based on linear regression (line is plotted without 95% CI). P_a_O_2_, arterial oxygen tension; SVRI, systemic vascular resistance index; PVD, perfused vessel density; CI, Cardiac index

The absolute values for all systemic hemodynamics at each study period are reported in Table 2. Hyperoxia resulted in a linear decrease in CI (Figure 1, *P*_slope_ = .009). The largest decrease occurred while breathing pure oxygen (max −10%, SD 10). This decrease was due to a similar reduction in HR (max −10%, SD 7, *P*_slope_ = .009), as stroke volume remained unchanged (*P*_slope_ = .75). MAP was not affected by hyperoxia (*P*_slope_ = .68). SVRI increased slightly over the entire P_a_O_2_ range (Figure 2) to a maximum of +7% (SD 8, *P*_slope_ = .009). On visual inspection, the SVRI data fitted a second order polynomial equation better than a linear one, although the Sy.x statistics were identical (8.1).

**Table 2:** Measurements

Period	Air	T1	T2	T3	Oxygen	*P* *
Intervention (n = 15)						
Target (kPa)	–	20 (1.5)	40 (1.5)	60 (1.5)	–	–
P_a_O_2_ (kPa)	14 (1.3)	20 (1.8)	39 (3.0)	57 (4.9)	73 (4.1)	–
F_I_O_2_ (%)	21 (0)	29 (2)	55 (3)	80 (5)	100 (0)	–
Arterial blood gas (n = 15)						
S_a_O_2_ (%)	98 (1)	99 (1)	100 (0)	100 (1)	100 (1)	<.001
P_a_CO_2_ (kPa)	4.7 (0.7)	4.8 (0.6)	4.6 (0.8)	4.8 (0.5)	4.8 (0.5)	.98
pH	7.4 (.04)	7.4 (.03)	7.5 (.05)	7.4 (.04)	7.4 (.03)	.51
Hb (mmol L^−1^)	8.6 (0.8)	8.5 (0.9)	8.6 (0.8)	8.6 (0.7)	8.6 (0.7)	.84
Glucose (mmol L^−1^)	6.3 (1.3)	6.1 (1.2)	6.0 (1.0)	5.7 (0.8)	5.6 (0.6)	.028
Lactate (mmol L^−1^)	1.0 (0.6)	0.9 (0.4)	0.9 (0.4)	0.8 (0.3)	0.7 (0.2)	.015
Microcirculation (n = 15)						
VD (n/mm)	8.0 (0.9)	8.0 (1.0)	8.0 (1.8)	7.6 (1.2)	6.9 (1.5)	.02
PVD (n/mm)	7.8 (1.0)	7.8 (1.0)	7.6 (1.6)	6.9 (1.0)	6.5 (1.5)	.001
IFD (n/mm)	0.07 (.09)	0.16 (.14)	0.30 (.31)	0.46 (.32)	0.23 (.27)	<.001
PPV (%)	98 (2.2)	97 (1.9)	96 (3.8)	91 (3.7)	93 (8.3)	<.001
MFI	3.0 (.09)	2.9 (.15)	2.9 (.22)	2.8 (.29)	2.7 (.31)	<.001
Hemodynamics (n = 14)						
HR (bpm)	64 (7)	62 (8)	60 (8)	58 (7)	58 (7)	.009
SVI (mL min^−1^m^−2^)	56 (7)	57 (7)	58 (8)	58 (7)	57 (8)	.75
CI (L min^−1^m^−2^)	3.6 (0.7)	3.5 (0.6)	3.4 (0.6)	3.3 (0.6)	3.3 (0.6)	<.001
SVRI (dyn·s cm^−5^ m^−2^)	2142 (359)	2199 (368)	2236 (426)	2317 (431)	2288 (408)	.009
MAP (mm Hg)	98 (16)	98 (14)	98 (15)	98 (13)	95 (13)	.68
DO_2_ (n = 14)						
C_a_O_2_ (mL/L)	192 (18)	194 (20)	200 (18)	206 (16)	208 (17)	<.001
DO_2_ (mL min^−1^m^−2^)	684 (141)	676 (132)	686 (133)	695 (141)	679 (145)	.83

Data are presented as mean (SD). VD, vessel density; PVD, perfused vessel density; PPV, proportion of perfused vessels; MFI, microvascular flow index; IFD, intermittent flow density; HR, heart rate; MAP, mean arterial pressure; SVI, stroke volume index; CI, cardiac index; SVRI, systemic vascular resistance index; C_a_O_2_, arterial oxygen content; DO_2_, oxygen delivery. *P‐values for slope.

### 3.4 Oxygen delivery

The oxygen content of arterial blood increased linearly to a maximum of +8% (SD 3, *P*_slope_ < .0001) when breathing pure oxygen (Table 2, Figure 1). Systemic DO_2_ index remained unaltered (*P*_slope_ = .83).

### 3.5 Sublingual microcirculation

**Figure 3:**
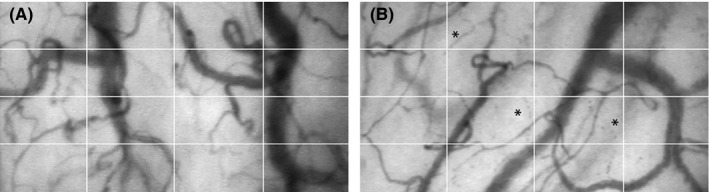
Sublingual microcirculation. Representative images of the sublingual microcirculation acquired with the SDF device. Compared to breathing air (A), oxygen supplementation (B) decreased overall VD (vessels crossing the white grid) and caused interrupted flow (asterisk). SDF, sidestream darkfield imaging; VD, vessel density

P_a_O_2_ reduced, in a dose‐dependent fashion, vascular density (VD) and perfused vascular density (PVD). Compared to measurements performed at baseline, VD changed with +1% (SD 13), 0% (SD 18), −4% (SD 12), and −13% (SD 17, *P = *.005) at a P_a_O_2_ of 20, 39, 57, and 73 kPa, respectively. Similarly, PVD changed with +0% (SD 15), −3% (SD 18), −11%(SD 13), and −15% (SD 18, *P *=* *.003). The data could be fitted with both a straight (*P*_slope_ < .0001) and a sigmoidal line. The standard deviation of the values around the regression line (Sy.x) was 13.6 and 13.7, respectively. On visual inspection, the sigmoidal curve was a better fit (Figure 2). The number of vessels showing intermittent flow increased linearly up to 57 kPa, but was then relatively reduced at 73 kPa. A representative image of the microcirculation while breathing room air is shown in Figure 3A. When breathing oxygen, the number of perfused vessels was reduced and blood flow became stagnant or intermittent, visible as “dotted” vessels in Figure 3B.

## 4 DISCUSSION

The main finding of this study is that in healthy volunteers, supplemental oxygen does not alter DO_2_, while sublingual PVD decreased in a sigmoidal fashion as P_a_O_2_ was increased stepwise from 14 up to 73 kPa. Hyperoxia decreased CI, by a reduction in HR rather than stroke volume, and increased SVRI. MAP was unchanged.

In this healthy volunteer population, the increase in C_a_O_2_ caused by an increase in P_a_O_2_ was negated by a simultaneous reduction in CI. Two previous studies in healthy volunteers on hemodynamic effects of oxygen showed a slight decrease in DO_2,_ 24, 34 while another showed no effect.^35^ In patients, a similar heterogeneity has been seen as DO_2_ was reduced in 2 studies,^22^, ^23^ but remained unaltered in 2 others.^17^, ^19^ An important conclusion from our study is that, in nonhypoxemic individuals, an intended increase in DO_2_ is not achieved by any level of normobaric oxygen supplementation.

We found a significant reduction in sublingual PVD simultaneous to a stepwise increase in P_a_O_2_ in this group of healthy individuals. Changes in the sublingual microcirculation in response to an increase in the F_I_O_2_ to 1.0 also occur in patients after coronary artery bypass surgery and in a cohort of mixed ICU patients (postcardiac arrest, neurological defects, polytrauma, sepsis).^18^, ^36^ Critical illness is associated with (regional) disturbances in microcirculatory perfusion.^37^ It is possible that systemic oxygen‐induced changes in blood flow further impair regional perfusion and cause a mismatch between DO_2_ and demand.^38^ For example, in an animal model for severe coronary artery stenosis, hyperoxia was found to exacerbate myocardial ischemia due to coronary vasoconstriction.^39^ In our study, we found no evidence for cellular hypoxia (as indicated by lactate), but this may be different in the critically ill with pre‐existing perfusion defects. Either way, in terms of oxygenation, there appears to be no clear advantage to supraphysiological oxygen tensions; in a best‐case scenario, there is no effect on DO_2_, and in a worst‐case, an unintended reduction.

This is the first study in which dose‐dependent effects of oxygen on the sublingual microcirculation are described. The relation between P_a_O_2_ and PVD could be fitted with both a linear and a sigmoidal curve. Both models performed similarly based on the standard deviation of errors in regression. However, the graphical presentation of the data pleads for a sigmoidal relation between P_a_O_2_ and PVD. Also, a nonlinear relation is corroborated by observations carried out in hamsters,^40^, ^41^ rats,^42^ and rabbits.^43^ In another study with healthy volunteers, a reduction in PVD of approximately 30%^32^ was found, which is much larger than the effect (of ~15%) found in our study. Discrepancies may be explained by differences in study design: prolonged exposure to hyperoxia (30 vs 10 minutes) and an acute exposure to pure oxygen (vs the gradual increase in our study). The SVR, which is an indicator of vasoconstriction, shows a sharp increase after 5‐10 minutes and then further increases by a small amount over the course of an hour in healthy volunteers^25^, ^44^ and postoperative critically ill patients.^17^ SVR does not show a larger increase when volunteers are acutely^16^, ^24^, ^26^, ^27^, ^45^, ^46^, ^47^, ^48^ or chronically exposed to oxygen.^15^, ^35^, ^49^, ^50^, ^51^ The difference in effect size may therefore be partially explained by the exposure time.

The exact mechanism behind the constrictive effect of high arterial oxygen tensions is currently unclear. Reactive oxygen species (ROS) are one possible candidate, considering that in vitro, as PO_2_ increases, so does the production of superoxide.^52^ Superoxide reacts heavily with the vasodilator nitric oxide, reducing its bioavailability and thereby causing vasoconstriction. This has been shown directly in porcine coronary arteries^53^ and indirectly in human studies; where the scavenging of ROS by infusion of high levels of vitamin C reduced or prevented hyperoxic constriction.^11^, ^12^, ^54^ However, the involvement of ROS is not found uniformly.^25^, ^55^ Intravital studies in hamsters, rat, and mice suggest that other pathways may be involved, including inactivation of calcium^56^, ^57^ and potassium channels^57^, ^58^ or the alteration of the metabolism of arachidonic acid.^59^, ^60^ For instance, high PO_2_ was found to decrease the activity of the enzyme cyclooxygenase, reducing the formation of dilating prostaglandins from arachidonic acid.^61^, ^62^, ^63^ Conversely, the production of the vasoconstrictor 20‐HETE from arachidonic acid by the CYP‐450 pathway was shown to be increased by hyperoxia.^64^, ^65^, ^66^ These observations are however not mutually exclusive, different mechanisms of hyperoxic vasoconstriction may be present depending on the vascular bed and/or species under investigation.

Our study shows a small imbalance between the reduction in PVD and SVRI. At higher P_a_O_2_s (>57 kPa), the fractional decrease in PVD was larger than the increase in SVRI. Based on the data from our study, we can only speculate why this is the case. One possible explanation is heterogeneity between microvascular beds: Capillary recruitment in the sublingual area may decrease, while recruitment in other areas/organs remains unaltered or increases. As a result, the effect of increasing P_a_O_2_ on the PVD in the sublingual microcirculation may be larger than on SVR. In anaesthetized dogs, hyperoxia has been shown to redistribute blood flow to the kidney, liver, and intestines, while blood flow to the myocardium, pancreas, and skeletal muscle decreases.^67^ The amount of redistribution may vary at different P_a_O_2_s. Another explanation is recruitment of arteriovenous shunts, which provide relatively less resistance to flow than smaller arterioles/capillaries and therefore mitigate the increase in SVR. Shunting may also explain the decrease in VO_2_ that is seen in some studies after oxygen administration.^17^, ^68^ A bypass of metabolic active tissue will elevate venous PO_2_, reducing the arteriovenous oxygen difference used to calculate VO_2_. However, we did not take venous blood samples to calculate VO_2_, as this was beyond the scope of our study.

In our study, the majority of hyperoxia‐induced changes became apparent above an oxygen tension of 20 kPa. From a hemodynamic and microcirculatory point of view, arterial oxygen tensions up to 20 kPa could therefore be suggested as “permissive hyperoxia.” Interestingly, the range of 10‐20 kPa is retrospectively associated with lower mortality in critically ill populations^5^, ^10^ and with improved organ function after cardiac arrest.^9^ Slight hyperoxia (up to a P_a_O_2_ of 20 kPa) may thus be beneficial, because its influence on perfusion is possibly negligible. However, even within this range, one prospective study in ICU patients showed reduced mortality in a conservative oxygen group (median P_a_O_2_ of 11.5 kPa) compared to a conventional oxygen group (median P_a_O_2_ of 13.5 kPa). Beside some methodological issues, it should be noted that the study was stopped prematurely due to slow inclusion and was therefore underpowered for a mortality endpoint.^69^, ^70^ Large randomized controlled studies in specific critically ill populations are required to determine whether slight hyperoxia is beneficial or not.

In healthy volunteers, supraphysiological arterial oxygen tensions, in the range of 14‐73 kPa, have no effect on systemic DO_2_; however, sublingual microcirculatory PVD decreased in a dose‐dependent fashion. Simultaneous with the increase in C_a_O_2_, cardiac output decreased due to a decline in HR rather than stroke volume. SVRI increased slightly, while MAP remained unaltered. All hemodynamic changes appear negligible up to a P_a_O_2_ of 20 kPa.

### 4.1 Limitations

Our study has several limitations. For one, we used a noninvasive measurement of systemic hemodynamics which is less precise than the gold standard thermodilution method. The true effect size of hyperoxia on systemic hemodynamics may therefore be different.

Second, we included a relatively low number of participants, but the study was adequately powered given the prepost study design.

Third, we performed the study in a single‐blind fashion due to the incremental oxygen exposure. This approach was chosen because of the possible residual effects of oxygen inhalation on hemodynamics for up to 30 minutes.^27^ The risk of operator bias was reduced by blinded analysis of the microvascular recordings and the use of a set time‐period for the averaging of hemodynamic variables.

Fourth, we did not include a control group that exclusively inhaled air during the study. This means we cannot exclude the possibility of time or comfort related changes in hemodynamics (eg, a reduction in HR due to increased comfort, rather than oxygen). However, we think it is highly unlikely that an increased level of comfort toward the end of the study is a factor in our results; all volunteers had ample time to adjust to the measurements before the start of the study and they showed no signs of anxiety at any time (eg, raised blood pressure). Our results on HR, MAP and stroke volume are in line with several other studies performed in healthy volunteers.^34^, ^35^, ^45^, ^49^, ^51^, ^71^, ^72^ HR and sublingual microcirculatory perfusion decreased dose‐dependently throughout the entire protocol; if there was no interaction with the intervention and the effect was solely due to increased comfort, we would have expected the effect to stabilize after an initial 20 or 30 minutes. Also, we are unaware of any mechanism that may link comfort with reduced sublingual perfusion. However, we cannot completely exclude a partial effect of comfort in our results; therefore, we advise future studies to include either a time control (eg, a group inhaling air only) or a phase with return to baseline (eg, air after oxygen exposure).

Fifth, the sublingual microcirculation may not be representative for other parts of the human body. However, we chose to investigate the sublingual area with SDF because of its noninvasive nature and has been used extensively in studies with the critically ill; it is a clinically relevant area as it is correlated with mortality and organ failure in patients with cardiogenic shock^73^ and sepsis^74^ and with postoperative complications after abdominal surgery.^75^ Also, the sublingual area is perfused directly from the carotis externa, making it very closely related to the central circulation.

## PERSPECTIVE

Despite decades of research into the cardiovascular effects of hyperoxia, the effects of clinically relevant high arterial oxygen tensions (hyperoxia) on systemic DO_2_ and sublingual microcirculatory perfusion are currently unknown.

In this arterial oxygen tension guided study, we found that in healthy volunteers, any level of normobaric oxygen supplementation has no effect on systemic DO_2_. Simultaneously, sublingual PVD was substantially decreased in the hyperoxic range of 20‐73 kPa.

Due to the prevalence of hyperoxia in critically ill patients, these findings warrant studies to determine whether hyperoxia exacerbates pre‐existing microcirculatory defects.

## CONFLICT OF INTEREST

The authors declare that they have no conflicting interests.
